# Ethical criteria for self-identifying societal risk associated with dangerous gain-of-function research

**DOI:** 10.1128/msphere.00607-25

**Published:** 2025-12-10

**Authors:** Patricia Delarosa, C. Matthew Sharkey, Kristjan Hollo

**Affiliations:** 1 Thriving Environments LLC., Oceanside, California, USA; 2 RAND Corporation 331135, Arlington, Virginia, USA; 3 Georgetown University 8368, Washington, DC, USA; Virginia-Maryland College of Veterinary Medicine, Blacksburg, Virginia, USA

**Keywords:** dangerous gain of function research, significant societal consequences, research proposal assessment

## Abstract

The 5 May 2025 executive order (EO) “Improving the safety and security of biological research” established a federal funding pause for dangerous gain-of-function (DGoF) research, defined as seeking certain experimental outcomes and deemed capable of resulting in significant societal consequences. These moves place institutional biosafety committees central in the identification and self-reporting of DGoF. The previous federal review for research anticipated to result in enhanced potential pandemic pathogens involved a multidisciplinary board, including a bioethicist. From our experience on those boards and based on the EO’s mandate to assess the significance of the societal consequences that might result from proposed DGoF research, we suggest a layered review process for the assessment of societal consequences to inform implementation of the EO. In the layered review, proposed research, initially identified based on anticipated experimental outcomes, is confirmed as DGoF through an assessment that is informed by ethical frameworks.

## PERSPECTIVE

The 5 May 2025 executive order (EO) “Improving the safety and security of biological research,” EO 14292, directs the government to suspend dangerous gain-of-function (DGoF) research until a revised or replaced high-consequence research policy is issued (1). Associated National Institutes of Health (NIH) notices from 7 May and 18 June (2, 3) direct awardees to identify and self-report DGoF. DGoF is research with any pathogen or toxin that seeks to achieve one or more of seven experimental outcomes that were previously included in DURC policies (4–6) and is further characterized as research whose outcomes “could cause significant societal consequences” (SSCs). Although institutional biosafety committees (IBCs) are familiar with identifying the experimental outcomes associated with dual use research of concern (DURC), SSC is a new criterion that has not been included in previous institutional research oversight policies. A research ethics review was part of the 2017 federal oversight of similar research proposals (7). This review followed the 2017 recommendations from the National Science Advisory Board for Biosecurity (NSABB) (8) that called for an analysis of risk to determine if the intended research benefits justified the public health risks associated with manipulation of a pathogen with increased transmissibility and virulence. The 2017 White House Office of Science and Technology Policy (OSTP) policy on the review and funding of research similar to DGoF called for an ethics review, whose criteria included non-maleficence, beneficence, justice, respect for persons, scientific freedom, and responsible stewardship (9).

Terms such as beneficence and justice are found in ethical and legal policy documents that may offer an initial path forward for the review of SSC. For scientific research, principal among these are the Belmont report (10) and the Biological Weapons Convention (BWC) (11); however, supporting policies such as the InterAcademy Partnership (IAP) Statement on Biosecurity (12) offer further guidance on responsible stewardship in science.

The Belmont report establishes the U.S. policy for ethics in research involving human subjects (10). It includes basic principles for biomedical and behavioral research. In part B, titled “Basic ethical principles,” it promotes ideas that are easily transferred to the discussion of societal consequences related to DGoF. Among these, the principle of beneficence requires that “[p]ersons are treated in an ethical manner, not only by respecting their decisions and protecting them from harm but also by making efforts to secure their well-being.” It further elaborates that “[t]he obligations of beneficence affect both individual investigators and society at large, because they extend both to particular research projects and to the entire enterprise of research. Investigators and members of their institutions are obliged to give forethought to the maximization of benefits and the reduction of risk that might occur from the research investigation. In the case of scientific research in general, members of the larger society are obliged to recognize the longer-term benefits and risks that may result from the improvement of knowledge and from the development of novel medical, psychotherapeutic, and social procedures.” In this passage, the terms “research enterprise” and “larger society” suggest that the obligation to maximize the benefits of research while reducing the risks is ethical requirements that extend beyond the scientific review of life science research. These ideas are not explicitly included in the current IBC review process (13), and although the 2017 federal review of enhanced potential pandemic pathogen research—which was similar in its scope to DGoF—included “larger societal” issues as related to public health (7), this concept was not extended to the “research enterprise” or to conformance with societal norms. The 2015–2023 NSABB reviews (14) were open to the public, a research ethicist sat on the NSABB board, and recommendations included ethical concepts from the Belmont report but as noted in recommendation 5.1 from the NSABB’s 2023 report (15), the OSTP policy was not fully consistent with this recommendation, as it did not provide guidance on the risk-benefit analysis. The consideration of “longer-term benefits and risks” by the NSABB was heavily focused on scientific advancements as the primary societal consideration, whereas the risks of the pathogen’s escaping containment were not central to their approach given existing biosafety policies and required containment practices (16).

The Belmont report also covers the idea of justice, described by the question, “[w]ho ought to receive the benefits of research and bear its burdens?” The seven research outcomes, now used to select research with the potential to be DGoF, describe enhancements to pathogenic or toxic traits that have the potential to cause epidemic or pandemic disease or pose unacceptable toxic dangers—to people, livestock, or crops. The dire societal consequences seen during the COVID-19 pandemic—in terms of human life and economic, educational, and social opportunities lost—should inform the consideration of SSC when assessing DGoF research; the COVID-19 experience frames the EO’s requirement for review of SSC as an ethical assessment review process. This is separate from the IBC’s purview of assessing the adequacy of biosafety practices. The assessment of SSC is founded on the assumption that biocontainment strategies may conceivably fail. The assessment must focus on whether the consequences of introducing a biological agent, whose properties are reasonably anticipated to have been modified to achieve the proposed experimental outcomes, are acceptable—in light of the anticipated benefits to society from the research. No previous research oversight framework has been fundamentally grounded in the anticipation that biocontainment practices can, and—with some non-zero probability—will, fail.

Part C of the Belmont report provides guidance on the benefit/risk analysis, emphasizing that “alternative ways of obtaining the benefits sought in the research” must be explored to counter “concern about the loss of substantial benefits that might be gained from research.”

The BWC prohibits life sciences research with no justification for prophylactic, protective, or other peaceful purposes, while also asserting that state parties retain the right to conduct research that is peaceful and stipulates that the prohibition should be designed to avoid hampering the economic or technical development of state parties (11). This document is internationally recognized and foundational to U.S. legal codes about the peaceful use of microorganisms (17). The idea that some life sciences research poses unacceptable risks to society and should not be carried out is recognized in the NSABB and Belmont reports and by the prohibition contained in the BWC (10, 11). Furthermore, the IAP statement—released in anticipation of a meeting of state parties in 2005—also stresses the importance of scientists identifying and reporting violations of those prohibitions (12). As the BWC is an international legal document, reflecting strong worldwide consensus against the misuse of the life sciences, it is likely that a federally mandated ethics review of DGoF that includes an assessment of the misuse criteria from the BWC could be extended to privately funded research, closing a recognized loophole that was inherent in past review mandates—which only covered federally funded research. We note that the legal authorities to extend the prohibitions against DGoF that pose SSC were requested in section 5 of the 5 May EO (1).

Calls for the assessment of ethics in gain-of-function research were identified as a critical need due to the unique potential that this type of research poses not only to occupational health but also public health (16). This type of review may have been more difficult to initiate in the past due to an established patchwork of biosafety and biosecurity regulatory documents that were all based on pathogen lists and traditional pathogen risk groups (4–6, 13, 18). Previously, high-consequence research oversight frameworks established the reasonably anticipated experimental outcomes included in the EO and NIH notices and asked awardees to assess those outcomes for experiments that manipulated defined categories of microbial agents (4–6). This limited the scope of covered research to a list of defined biological select agents and toxins or risk group-3 and -4 agents, and this scope aligned with internal institutional processes focused on occupational health and safety of researchers in biosafety level 3 and 4 laboratories. Opening the review aperture to apply the reasonably anticipated research effects to all pathogens and toxins shifts the review process from an agent- to outcome-focused assessment, which is more appropriate for the review and oversight of enhanced agents whose transmissibility or virulence may be enhanced in a manner not previously studied, especially given emerging capabilities in biodesign (19, 20). The EO identifies SSC—an ethical assessment of anticipated benefits and potential bad outcomes—as the final filter to stratify and prioritize risks within the pool of potential DGoF research. This new approach represents an opportunity for life sciences researchers to develop criteria to establish the boundaries for SSC to ensure the consistent, transparent, and defensible application of these policies. Clear SSC assessment criteria—grounded in ethical considerations—are fundamental to public transparency and the creation of trust between the life sciences research enterprise and the public. Life science researchers must interpret ethical and legal guidelines to define criteria for the evaluation of SSC. Depending upon scope, this will encompass a multidisciplinary ethics review, which may be worthy of its own separate review board. To alleviate shortcomings that challenged the implementation of the criteria outlined in the EO and NIH memoranda, we recommend that the life science research community begin to develop uniform SSC assessment criteria for life science research. Recently, one of the authors participated in drafting a framework that may be useful in further refining the SSC assessment (21).
